# Human Betacoronavirus OC43 Interferes with the Integrated Stress Response Pathway in Infected Cells

**DOI:** 10.3390/v16020212

**Published:** 2024-01-31

**Authors:** Stacia M. Dolliver, Caleb Galbraith, Denys A. Khaperskyy

**Affiliations:** Department of Microbiology and Immunology, Faculty of Medicine, Dalhousie University, Halifax, NS B3H 4R2, Canada

**Keywords:** betacoronavirus OC43, integrated stress response, PERK, PKR, GADD34, ATF4

## Abstract

Viruses evolve many strategies to ensure the efficient synthesis of their proteins. One such strategy is the inhibition of the integrated stress response—the mechanism through which infected cells arrest translation through the phosphorylation of the alpha subunit of the eukaryotic translation initiation factor 2 (eIF2α). We have recently shown that the human common cold betacoronavirus OC43 actively inhibits eIF2α phosphorylation in response to sodium arsenite, a potent inducer of oxidative stress. In this work, we examined the modulation of integrated stress responses by OC43 and demonstrated that the negative feedback regulator of eIF2α phosphorylation GADD34 is strongly induced in infected cells. However, the upregulation of GADD34 expression induced by OC43 was independent from the activation of the integrated stress response and was not required for the inhibition of eIF2α phosphorylation in virus-infected cells. Our work reveals a complex interplay between the common cold coronavirus and the integrated stress response, in which efficient viral protein synthesis is ensured by the inhibition of eIF2α phosphorylation but the GADD34 negative feedback loop is disrupted.

## 1. Introduction

Eukaryotic cells can rapidly respond to adverse conditions by activating specific stress response mechanisms. One of the conserved signaling pathways activated by various types of stress, including viral infections, is the integrated stress response (ISR) [1]. Its defining feature is the phosphorylation of the alpha subunit of the eukaryotic translation initiation factor 2 (eIF2α) on Serine-51, which causes the transient inhibition of bulk protein synthesis to conserve energy and minimize damage [2]. In mammals, there are four eIF2α kinases that are activated by different types of stress: heme-regulated inhibitor (HRI) kinase is activated by oxidative stress and heat shock [3,4], general control nonderepressible 2 (GCN2) kinase is activated by amino acid starvation or ultraviolet damage [5,6]; double stranded RNA (dsRNA)-activated protein kinase (PKR) is activated by dsRNA [7]; and PKR-like endoplasmic reticulum (ER) kinase (PERK) is activated by ER stress [8]. Although PKR is the kinase primarily dedicated to sensing viral dsRNA replication intermediates, other eIF2α kinases can also be activated by viral infections that perturb cellular homeostasis [9]. Since viruses rely on host machinery for their protein synthesis, they must evolve mechanisms to block or bypass the ISR to ensure efficient replication.

Upon phosphorylation, eIF2α stably binds the guanine exchange factor eIF2B, which prevents the regeneration of the translation initiation-competent GTP-bound form of eIF2 [2]. This stalls bulk translation initiation, while simultaneously causing the increased translation of a subset of messenger RNAs (mRNAs), some of which contain short upstream open reading frames (uORFs) in front of ORFs that encode stress response factors [10,11]. The depletion of initiation-competent eIF2 complexes increases leaky scanning, or uORF bypass by the 48S preinitiation complex, which promotes the translation of downstream ORFs [2]. Activating transcription factor 4 (ATF4) is one of the important ISR factors preferentially translated via uORF bypass [12]. This transcriptional activator induces the expression of additional stress response genes, many of which are also encoded by uORF-containing mRNAs. They include another transcription factor C/EBP homologous protein (CHOP) and its downstream target growth arrest and DNA damage-inducible protein 34 (GADD34) [12]. GADD34 (also named protein phosphatase 1 regulatory subunit 15A, PPP1R15A) complexes with protein phosphatase 1 (PP1) and recruits it to dephosphorylate eIF2α [13]. Thus, the main function of GADD34 in the ISR is the negative feedback that terminates phospho-eIF2α mediated translation arrest.

Coronaviruses (CoVs) are a family of enveloped single-stranded positive-sense RNA viruses. There are seven CoVs known to infect humans, presenting from common colds to more severe and sometimes fatal respiratory disease [14]. Common cold coronaviruses include HCoV-OC43 (OC43) and HCoV-229E, which have been circulating in the human population for decades, as well as more recently identified HCoV-NL63 and HCoV-HKU1 [15]. The most recent pathogenic CoV, the severe acute respiratory syndrome coronavirus 2 (CoV2), emerged in 2019 and became the cause of the devastating pandemic of coronavirus disease 2019 (COVID-19). CoV2, as well as OC43 and HCoV-HKU1, belong to the Betacoronavirus genus [14,15]. In recent years, OC43 emerged as the model Betacoronavirus for studying viral replication and testing the candidate anti-CoV drugs because, unlike CoV2, it is considered safer and can be handled in a more accessible containment level 2 environment [16,17,18,19,20].

The OC43 virion has five structural proteins. Membrane (M) and envelope (E) proteins are responsible for viral particle formation. Nucleocapsid (N) is an RNA-binding protein that coats the viral genome. There are also two surface glycoproteins: hemagglutinin esterase (HE) and spike (S) [15]. S is a receptor binding protein that binds sialic acid on the cell surface [21] and mediates viral entry [14]. Once the virus enters a host cell, its large 30 kb positive sense capped and polyadenylated RNA genome associates with host translation machinery to initiate the synthesis of viral non-structural proteins (Nsps) involved in genome replication, subgenomic mRNA production, and the inhibition of host antiviral responses [14]. The replication of the viral genome in the cytoplasm can generate double-stranded RNA (dsRNA) intermediates which may activate cytoplasmic sensors, including retinoic acid-inducible gene I (RIG-I), melanoma differentiation-associated gene 5 (MDA5), and dsRNA-activated protein kinase (PKR) [7,22]. However, in CoV-infected cells, dsRNA is largely shielded inside double membrane replication transcription compartments that exclude and limit the activation of cytosolic sensors [14,23]. These double membrane compartments are derived from the endoplasmic reticulum (ER) of the host cell by the concerted action of viral Nsp3, Nsp4, and Nsp6 proteins [24,25]. Newly generated viral sub-genomic mRNAs that encode structural proteins are released from these compartments through specific crown-shaped pores and then associate with either free or ER-associated ribosomes in the cytoplasm to be translated [26].

Genomic RNA and sub-genomic mRNAs of coronaviruses are capped and polyadenylated and therefore resemble host mRNAs in their translation initiation mechanism, which is sensitive to the disruption of the cap-directed assembly of the translation preinitiation complex or eIF2α phosphorylation [27,28]. Therefore, coronaviruses must limit ISR to ensure robust protein synthesis and replication. Our previous work revealed that OC43 infection actively inhibits the phosphorylation of eIF2α [29]. In our cell culture infection model, very little eIF2α phosphorylation was detected in infected cells, and when they were treated with a potent inducer of eIF2α phosphorylation sodium arsenite (As), they had significantly reduced levels of p-eIF2α compared to mock-infected cells [29]. As treatment induces oxidative stress and activates HRI kinase [4]. This usually causes very strong eIF2α phosphorylation and the formation of stress granules (SGs), cytoplasmic condensates of polysome-free messenger ribonucleoprotein complexes that accumulate following the inhibition of translation initiation [9]. OC43 infection also inhibits the formation of As-induced stress granules (SGs) [29].

In the present work, we aimed to further characterize the modulation of ISR by OC43. We demonstrate that the OC43 infection of human embryonic kidney 293A cells strongly induces GADD34, the negative regulator of ISR. Although OC43 infection causes ER stress and the activation of PERK, which negatively affects viral protein synthesis and replication, PERK-mediated ATF4 production is not responsible for GADD34 upregulation in infected cells. Remarkably, we also show that OC43-induced GADD34 protein does not function in mediating increased eIF2α dephosphorylation.

## 2. Materials and Methods

### 2.1. Cells and Viruses

Human embryonic kidney (HEK) 293A cells and green monkey kidney (Vero) cells were purchased from American Type Culture Collection (ATCC, Manassas, VA, USA) and cultured in Dulbecco’s modified Eagle’s medium (DMEM) supplemented with heat-inactivated 10% fetal bovine serum (FBS) and 2 mM L-glutamine (all purchased from Thermo Fisher Scientific (Thermo), Waltham, MA, USA). Human betacoronavirus OC43 was purchased from ATCC. Initial virus stocks were generated in Vero cells as described in [29]. Subsequent OC43 passages for the expansion of stocks were undertaken in 293A cells. The 293A cells were infected at the multiplicity of infection (MOI) <0.1 for 1 h in serum-free DMEM, and then the inoculum was replaced with DMEM supplemented with 1% FBS + 0.5% BSA and incubation continued at 37 °C. At 48 h post infection (hpi), the viral supernatant was harvested, cleared by centrifugation at 2500× *g* for 5 min, and then aliquoted and stored at −80 °C. Virus titers were determined by 50% tissue culture infectious dose (TCID50) assay in 293A cells in the same infection media as above and calculated using the Spearman–Kärber method.

### 2.2. Cell Treatments

For poly(I:C) transfection, 1.5 µL of FuGENE HD reagent (Promega, Madison, WI, USA) was combined with 0.5 µg of low MW poly(I:C) (InvivoGen, San Diego, CA, USA) in 50 µL Opti-MEM-I (Gibco, Waltham, MA, USA) for 5 min and then added directly to media for each 20 mm well of cells. Cells were returned to a 37 °C incubator for 2 h. To induce eIF2α phosphorylation, sodium arsenite (As) was added to the media to a final concentration of 500 µM and cells were returned to the 37 °C incubator for 50 min; thapsigargin (Tg) was added to the media to a final concentration of 1 µM and cells were returned to the 37 °C incubator for 1 h or 4 h, as indicated. The integrated stress response inhibitor (ISRIB, final concentration 200 nM) or the Inositol-requiring enzyme 1 (IRE1) inhibitor 4µ8C (concentration range 5–25 µM) was added to the culture media and cells were returned to the 37 °C incubator for 23 h (for infection experiments) or the length of Tg treatment, as indicated. All compounds and inhibitors were purchased from Millipore Sigma (Oakville, ON, Canada).

### 2.3. Virus Infections

The 75–80% confluent monolayers of 293A cells grown on 20 mm wells of 12-well cluster dishes with or without glass coverslips were washed briefly with PBS and 300 µL of virus inoculum diluted to the specified MOI in 1% FBS DMEM. Cells were placed at 37 °C for 1 h, with manual horizontal shaking every 10–15 min. Then, virus inoculum was aspirated from cells, cells were washed briefly with PBS, and 1 mL of fresh 1% FBS DMEM was added to each well and cells were returned to 37 °C until the specified time post-infection.

### 2.4. Gene Silencing

For GADD34 silencing, 293A cells were transfected with Ambion Silencer Select siRNAs (siGADD34#1 (s24269) and siGADD34#2 (s24268)) using Lipofectamine RNAiMAX (Invitrogen, Waltham, MA, USA) according to manufacturer’s protocol and used in infection/treatment experiments at 48 h post-transfection. For non-targeting siRNA control, cells were transfected with Silencer Select Negative Control #2 siRNA (siNT; Ambion, #4390846). For ATF4 silencing, shRNA inserts targeting human ATF4 gene (shATF4-1, target sequence catgatccctcagtgcataaa; shATF4-2, target sequence cctaggtctcttagatgatta) or control human interleukin 2 (IL2) that is not expressed in epithelial cells (shIL2, target sequence gctacctattgtaactattat) were cloned into pLKO.1-TRC vector (Addgene plasmid #10878, a gift from David Root, [30]). For PERK silencing, a guide RNA insert targeting human PERK (sgRNA sequence gaatataccgaagttcaaag) was cloned with the lentiCRISPR-v2 vector (Addgene plasmid #52961, a gift from Feng Zhang, [31]). The shRNAs and gRNA were designed using Broad Institute GPP Web Portal tools (https://portals.broadinstitute.org/gpp/public/). Then, 293A cells were transduced with lentiviruses generated with the above vectors at an MOI of 1.0, and stably transduced cells were selected with 1 μg/mL puromycin for 48 h. Resistant cells were seeded onto 12-well cluster dishes and used in experiments the following day.

### 2.5. Western Blotting

Whole-cell lysates were prepared by the direct lysis of PBS-washed cell monolayers with 1× Laemmli sample buffer (50 mM Tris-HCl pH 6.8, 10% glycerol, 2% sodium dodecyl sulphate (SDS), 100 mM DTT, 0.005% Bromophenol Blue). Lysates were immediately placed on ice, homogenized by passing through a 21-gauge needle, and stored at −20 °C. Aliquots of lysates thawed on ice were incubated at 95 °C for 3 min, cooled on ice, separated using denaturing polyacrylamide gel electrophoresis, transferred onto polyvinylidene fluoride (PVDF) membranes using Trans Blot Turbo Transfer System with RTA Transfer Packs (Bio-Rad Laboratories, Hercules, CA, USA) according to the manufacturer’s protocol, and analyzed by immunoblotting using antibody-specific protocols. Antibodies to the following targets were used: ATF4 (1:1000; rabbit, Abcam, ab270980); β-actin (1:2000; HRP-conjugated, mouse, Santa Cruz, sc-47778); eIF2α (1:1000; rabbit, Cell Signaling, #5324); GADD34 (1:1000; mouse, Proteintech, #10449-1-AP); OC43 N (1:1000; mouse, Millipore, MAB9012); OC43 Spike (1:1000; rabbit, Cusabio, CSB-PA336163EA-1HIY); PERK (1:1000; rabbit, Cell Signaling, #5683); phospho-S51-eIF2α (1:1000; rabbit, Cell Signaling, #3398); phospho-T451-PKR (1:1000; rabbit, Epitomics, 2283-1); PKR (1:1000; mouse, Santa Cruz, sc-6282); and XBP1s (1:1000; mouse, Cell Signaling, #12782). For band visualization, HRP-conjugated anti-rabbit IgG (goat, Cell Signaling, #7074) or anti-mouse IgG (horse, Cell Signaling, #7076) were used with Clarity Western ECL Substrate on the ChemiDoc Touch Imaging System (Bio-Rad Laboratories). For analyses of phospho-eIF2α band intensities, Western blot signals were quantified using Bio-Rad Image Lab 5.2.1 software and values normalized to the total eIF2α bands for each lane.

### 2.6. Immunofluorescence Microscopy

Cell fixation, methanol permeabilization, and immunofluorescence staining were performed according to the procedure described in [32]. Primary antibody staining was performed overnight at +4 °C with antibodies to OC43 N (1:500; mouse, Millipore, MAB9012) and/or TIAR (1:1000; rabbit, Cell Signaling, #8509). Alexa Fluor-conjugated secondary antibodies used were donkey anti-mouse IgG AF488 (Invitrogen, Burlington, Canada, A21202) and donkey anti-rabbit IgG AF555 (Invitrogen, A31572). Where indicated, nuclei were stained with Hoechst 33342 dye (Invitrogen, H3570). Slides were mounted with ProLong Gold Antifade Mountant (Thermo Fisher, Burlington, Canada) and imaged using a Zeiss AxioImager Z2 fluorescence microscope and Zeiss ZEN 2011 software.

### 2.7. RNA Isolation and RT-QPCR

Total RNA was isolated from cells using an RNeasy Plus Mini kit (Qiagen, Toronto, Canada) according to the manufacturer’s protocol and 250 ng of RNA was used to synthesize cDNA using qScript cDNA SuperMix (Quanta). Quantitative PCR amplification was performed using PerfeCTa SYBR Green PCR master mix (Quanta) with the specific primers listed below: 18S–Left: cgttcttagttggtggagcg, Right: ccggacatctaagggcatca; ACTB–Left: catccgcaaagacctgtacg, Right: cctgcttgctgatccacatc; GADD34–Left: ctcagcgccca-gaaac, Right: ggaaatggacagtgaccttc; GAPDH–Left: gagtcaacggatttggtcgt, Right: ttgattttggagggatctcg; and OC43-nsp15–Left: atggcgtagtggtggacaag, Right: actcccaggctgtcgaattg. Relative target levels were determined using the ΔΔCt method with normalization to 18S.

## 3. Results

### 3.1. The Targeting Subunit of eIF2α Holophosphatase GADD34 Is Strongly Upregulated in OC43-Infected Cells

**Figure 1:**
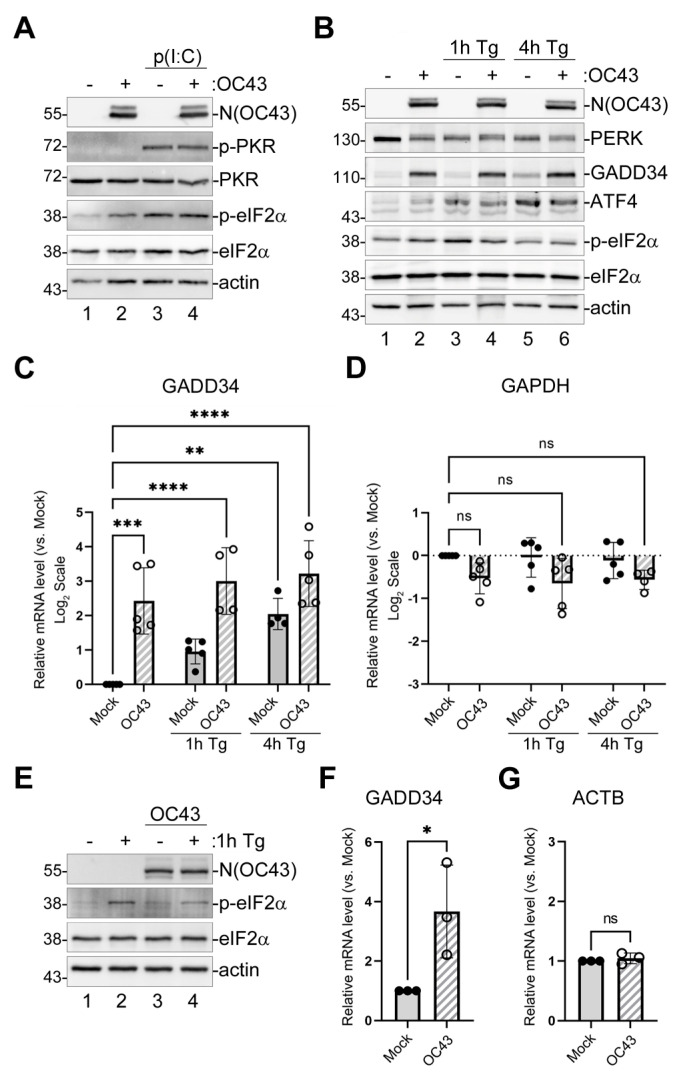
OC43 strongly induces GADD34 expression. 293A cells were infected with OC43 at MOI = 1.0. (**A**) Western blot analysis of lysates from mock- or OC43-infected cells collected at 24 h post-infection (hpi) with or without poly(I:C) transfection at 22 hpi (2 h poly(I:C) treatment). (**B**) Western blot analysis of lysates from mock- or OC43-infected cells collected at 24 hpi with or without Tg treatment for 1 or 4 h prior to harvesting. (**C**,**D**) Total RNA was isolated from mock- and infected cells treated as indicated and levels of GADD34 (**C**) and GAPDH (**D**) transcripts were determined by RT-QPCR. (**E**–**G**) Vero cells were infected with OC43 at MOI = 1.0. (**E**) Western blot analysis of lysates from mock- or OC43-infected Vero cells collected at 24 hpi with or without Tg treatment for 1 h prior to harvesting. (**F**,**G**) Total RNA was isolated from mock- and infected Vero cells and levels of GADD34 (**F**) and ACTB (**G**) transcripts were determined by RT-QPCR. On all plots, values were normalized to 18S and expressed as fold changes relative to mock untreated cells. Each data point represents an independent biological replicate (N ≥ 3). Error bars = standard deviation. Two-way ANOVA and Dunnett’s multiple comparisons tests were performed in C and D, and Student’s *t*-Test in (**F**,**G**) to determine statistical significance (****, *p*-value < 0.0001; ***, *p*-value < 0.001; **, *p*-value < 0.01; *, *p*-value < 0.05; ns, non-significant).

Our previously published analyses of eIF2α phosphorylation in OC43-infected cells showed that at 24 and 48 h post-infection (hpi), OC43 causes a small but consistent increase in p-eIF2α levels [29]. Therefore, to begin characterizing the effects of the virus on ISR, we wanted to test the activation status of host factors upstream and downstream of phospho-eIF2α in the pathway. Among the eIF2α kinases, we focused on PKR because of its primary function in recognition of viral dsRNA replication intermediates, and PERK, because coronaviruses are known to induce ER stress [33,34]. We infected 293A cells with OC43 at a multiplicity of infection (MOI) = 1.0 and analyzed the phosphorylation statuses of PKR, PERK, and eIF2α at 24 hpi using Western blotting. For positive controls, we used transfection with polyinosinic-polycytidylic acid (poly(I:C)) dsRNA mimic for PKR activation and treatment with the sarcoendoplasmic reticulum calcium ATPase (SERCA) inhibitor thapsigargin (Tg) for the induction of ER stress and the activation of PERK (Figure 1A,B). In both mock- and OC43-infected cells, poly(I:C) transfection induced the activation of PKR (as measured by phosphorylation at Threonine-451) and strong eIF2α phosphorylation, while no phospho-PKR signal could be detected in untransfected mock- or OC43-infected cells (Figure 1A). These results indicate that in our infection model, OC43 successfully blocks the detection of its dsRNA molecules by PKR. By contrast, virus infection caused the activation of PERK (as indicated by the appearance of an upstream shifted band corresponding to phosphorylated PERK), similar to the activation observed in mock-infected cells upon 1 h Tg treatment (Figure 1B, lanes 1–3). As expected, 4 h Tg treatment led to the induction of ATF4 and GADD34 and decreased phospho-eIF2α in both mock- and OC43-infected cells (Figure 1B, lanes 5,6). However, even without Tg treatment, OC43 infection strongly induced GADD34 to levels exceeding those observed after 4 h Tg treatment in mock cells (Figure 1B, compare lanes 2 and 5). To test if GADD34 protein accumulation was caused by increased mRNA levels, we isolated total RNA from mock- and OC43-infected cells and performed a reverse transcription–quantitative polymerase chain reaction (RT-QPCR) analysis of the GADD34 transcript. This analysis showed that, similar to 4 h Tg treatment, OC43 infection caused the strong upregulation of GADD34 mRNA levels (Figure 1C). Control GAPDH transcript was not significantly affected by Tg treatment or infection (Figure 1D). To confirm minimal eIF2α phosphorylation and strong GADD34 upregulation by OC43 infection in a different cell type, we used Vero cells. Similar to the infection of 293A cells, OC43 caused a small increase in eIF2α phosphorylation in Vero cells and decreased the maximal levels of phospho-eIFα upon Tg treatment (Figure 1E). The virus also caused the significant upregulation of GADD34 mRNA levels in Vero cells (Figure 1F,G), confirming that this phenotype is not specific to the 293A infection model.

### 3.2. The Activation of the Integrated Stress Response Pathway Is Not Responsible for GADD34 Induction in OC43-Infected Cells

**Figure 2:**
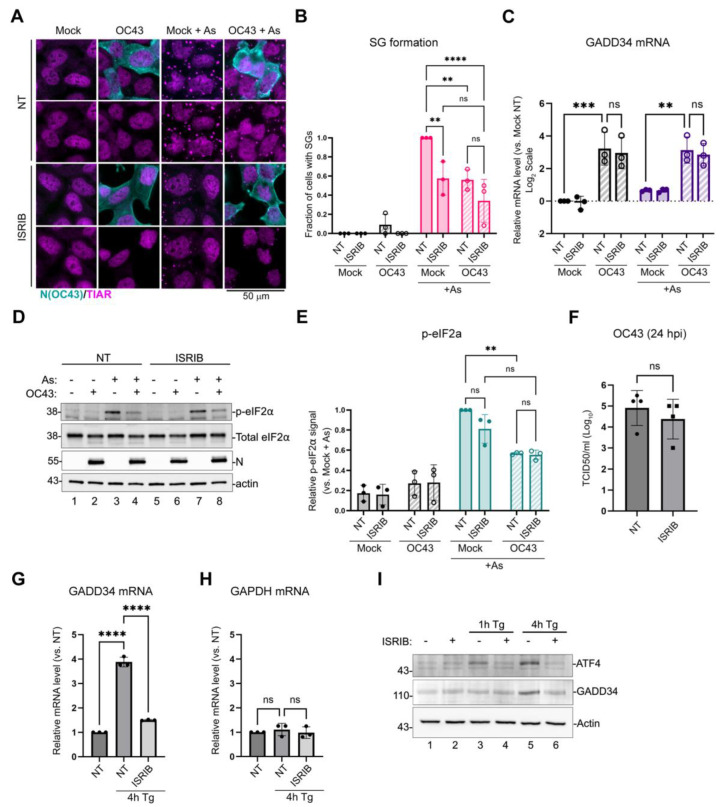
Integrated stress response inhibitor does not prevent GADD34 upregulation or the inhibition of eIF2α phosphorylation by OC43. The 293A cells were infected with OC43 at MOI = 1.0 and treated with ISRIB at 1 hpi or left untreated. At 23 hpi, p-eIF2α phosphorylation and SG formation was induced by the sodium arsenite treatment (+As). (**A**) SG formation in infected and mock-infected cells was analyzed using immunofluorescence staining for SG marker TIAR (magenta). Infected cells were identified by staining for nucleoprotein (N(OC43), teal). (**B**) SG formation was quantified from immunofluorescence staining images represented in (**A**). (**C**) Total RNA was isolated from mock- and infected cells treated as indicated and levels of GADD34 transcripts were determined by RT-QPCR. Values were normalized to 18S and expressed as fold changes relative to mock untreated cells. (**D**) Western blot analysis of lysates from mock- or OC43-infected cells treated as indicated. (**E**) Relative level of eIF2α phosphorylation was quantified from Western blot images represented in (**D**). (**F**) Production of infectious virions in untreated and ISRIB-treated cells was measured in cell supernatants at 24 hpi. (**G**–**I**) ISRIB blocks Tg-induced ATF4 and GADD34 expression in 293A cells. Uninfected cells were treated with Tg with or without the addition of ISRIB as indicated. (**G**,**H**) Levels of GADD34 (**G**) and GAPDH (**H**) transcripts were determined by RT-QPCR. Values were normalized to 18S and expressed as fold changes relative to mock untreated cells. (**I**) Western blot analysis of lysates from cells treated as indicated. For all plots, each data point represents an independent biological replicate (N ≥ 3). Error bars = standard deviation. Two-way ANOVA and Tukey multiple comparison tests or an unpaired *t*-Test (panel F only) were performed to determine statistical significance (****, *p*-value < 0.0001; ***, *p*-value < 0.001; **, *p*-value < 0.01; ns, non-significant).

Next, we wanted to determine whether ISR activation by OC43 was responsible for GADD34 upregulation and, consequently, the inhibition of As-induced eIF2α phosphorylation. To block phospho-eIF2α-mediated changes in translation initiation, we treated mock- and OC43-infected cells with ISR inhibitor (ISRIB, [35]). ISRIB does not affect eIF2α phosphorylation, but blocks its effects on the regeneration of the initiation-competent GTP-bound form of eIF2 and prevents translation arrest [36]. In our infection model, ISRIB treatment did not alter the inhibition of As-induced SG formation by OC43, while it significantly decreased As-induced SG formation in mock-infected cells, as expected (Figure 2A,B). It also did not affect OC43-mediated GADD34 induction (Figure 2C), the inhibition of As-induced eIF2α phosphorylation by OC43 (Figure 2D,E), or virus replication (Figure 2F). To test whether the ISR-dependent ATF4 and GADD34 induction pathway is intact in 293A cells, we verified the effects of ISRIB on Tg-induced ISR in this model. As expected, the Tg-mediated induction of ATF4 and GADD34 was blocked by ISRIB (Figure 2G–I). Taken together, these results show that neither GADD34 induction nor the inhibition of eIF2α phosphorylation by OC43 are due to ISR activation by the virus.

**Figure 3:**
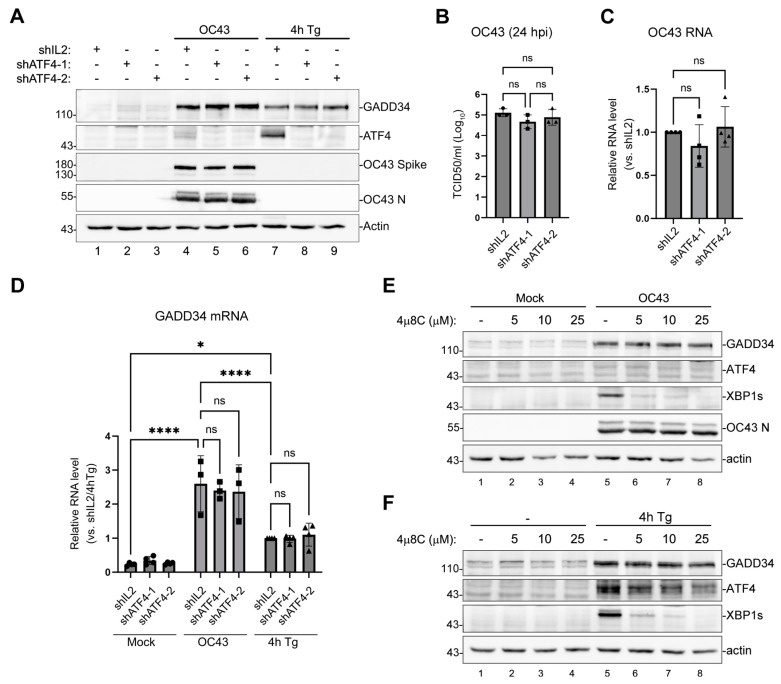
The upregulation of GADD34 expression by OC43 is ATF4-independent. (**A**–**D**) Cells transduced with lentiviruses encoding shRNAs against ATF4 (shATF4-1 and shATF4-2) or control IL-2 shRNA (shIL2) were infected with OC43 at multiplicity of infection (MOI) = 1.0 for 24 h or were treated with Tg for 4 h. (**A**) Western blot analysis of lysates from cells treated as indicated. (**B**) Production of infectious virions was measured in cell supernatants at 24 hpi. (**C**) Total RNA was isolated from infected cells and levels of OC43 genomic RNA were determined by RT-QPCR. Values were normalized to 18S and expressed as fold changes relative to shIL2 cells. (**D**) Levels of GADD34 transcripts in mock-infected, OC43-infected, and Tg-treated cells were determined by RT-QPCR. (**E**,**F**) IRE1 inhibition does not affect GADD34 upregulation in OC43-infected or Tg-treated cells. Western blot analysis of cells treated with the indicated concentrations of IRE1 inhibitor 4µ8C. (**E**) Mock- or OC43-infected cells analysed at 24 hpi. (**F**) Untreated (-) and Tg-treated cells. For all plots, each data point represents an independent biological replicate (N ≥ 3). Error bars = standard deviation. Two-way ANOVA and Tukey multiple comparisons tests were carried out to determine statistical significance (****, *p*-value < 0.0001; *, *p*-value < 0.05, ns, non-significant).

The principal upstream transcription factor induced upon eIF2α phosphorylation by PERK is ATF4. To confirm that ATF4 is not responsible for the transcriptional upregulation of GADD34 in OC43-infected cells, we silenced its expression using two lentivirus-driven shRNA constructs. As a non-targeting control, we used shRNA against IL2, the cytokine that is not expressed in epithelial cells. Both shRNA constructs were effective in decreasing Tg-induced ATF4 expression to nearly undetectable levels in uninfected cells, as well as OC43-induced ATF4 expression (Figure 3A). At the same time, neither shRNA affected the induction of GADD34 protein by OC43 infection or Tg treatment (Figure 3A). ATF4 silencing also did not affect OC43 protein accumulation, virus replication, or GADD34 mRNA induction (Figure 3B–D). In a classical ISR pathway, ATF4 induces the CHOP transcription factor, which then induces GADD34. However, GADD34 can be induced by other transcription factors [37,38,39], and in addition to PERK, Tg and OC43 can activate other ER stress sensors. One ER stress sensor that is strongly activated by OC43 is inositol-requiring enzyme 1 (IRE1) [33]. IRE1 activation causes the non-canonical cytoplasmic splicing of mRNA-encoding transcription factor X-box binding protein 1 (XBP1), leading to the synthesis of XBP1s, an activator of transcription produced from spliced XBP1 mRNA [40]. Although the IRE1/XBP1s pathway has not been shown to upregulate GADD34, we tested whether IRE1 inhibition would perturb GADD34 expression in OC43-infected cells. As reported previously in another model [33], OC43 infection induced XBP1s in 293A cells (Figure 3E). The addition of specific IRE1 inhibitor 4µ8C inhibited XBP1s’ induction triggered by OC43 infection or Tg treatment in a concentration-dependent manner (Figure 3E,F), but did not affect GADD34 induction.

### 3.3. The PERK Arm of the Unfolded Protein Response Negatively Affects OC43 Protein Synthesis and Replication

**Figure 4:**
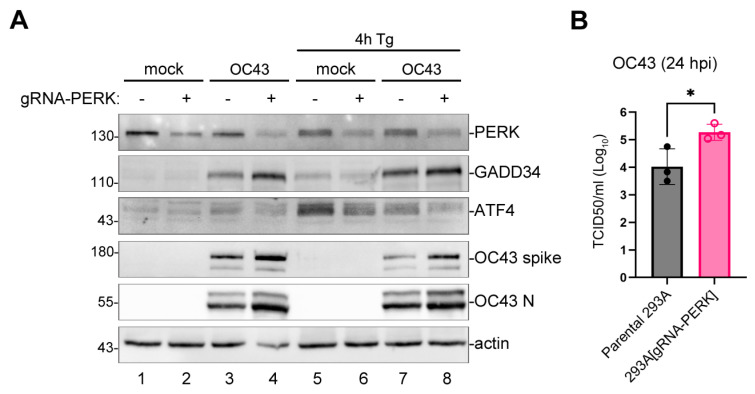
The silencing of PERK enhances OC43 protein synthesis and infectious virion production. The 293A cells transduced with lentivirus encoding Cas9 and guide RNA targeting PERK (gRNA-PERK) or parental untransduced cells were mock- or OC43-infected at MOI = 1.0. (**A**) Western blot analysis of cell lysates collected at 24 hpi. (**B**) The production of infectious virions was measured in cell supernatants at 24 hpi. Each data point represents an independent biological replicate (N = 3). Error bars = standard deviation. Student’s t-Test was performed to determine statistical significance (*, *p*-value < 0.05).

Having determined that OC43 infection activates PERK, we wanted to determine what role PERK plays in virus replication. First, we silenced PERK expression in 293A cells by transduction with the lentiviral-vector-encoding CRISPR/Cas9 system with guide RNA targeting PERK (gRNA-PERK). After 2 days, transduced cells expressed measurably lower PERK levels compared to parental untransduced cells (Figure 4A). When transduced cells were infected with OC43, they accumulated more viral proteins and GADD34 compared to parental cells (Figure 4A, lanes 3,4). Consistent with PERK being an upstream regulator of ATF4 expression, when transduced cells were treated with Tg, they induced lower levels of ATF4 protein compared to parental cells (Figure 4A, lanes 5,6). A similar effect was also observed in OC43-infected cells (Figure 4A, lanes 7,8). This suggests that PERK is not required for GADD34 upregulation by OC43 and that PERK-mediated ISR may have an antiviral role. Indeed, compared to parental cells, gRNA-PERK transduced cells generated significantly higher infectious virus titers (Figure 4B).

### 3.4. OC43-Induced GADD34 Does Not Contribute to Decreased eIF2α Phosphorylation

**Figure 5:**
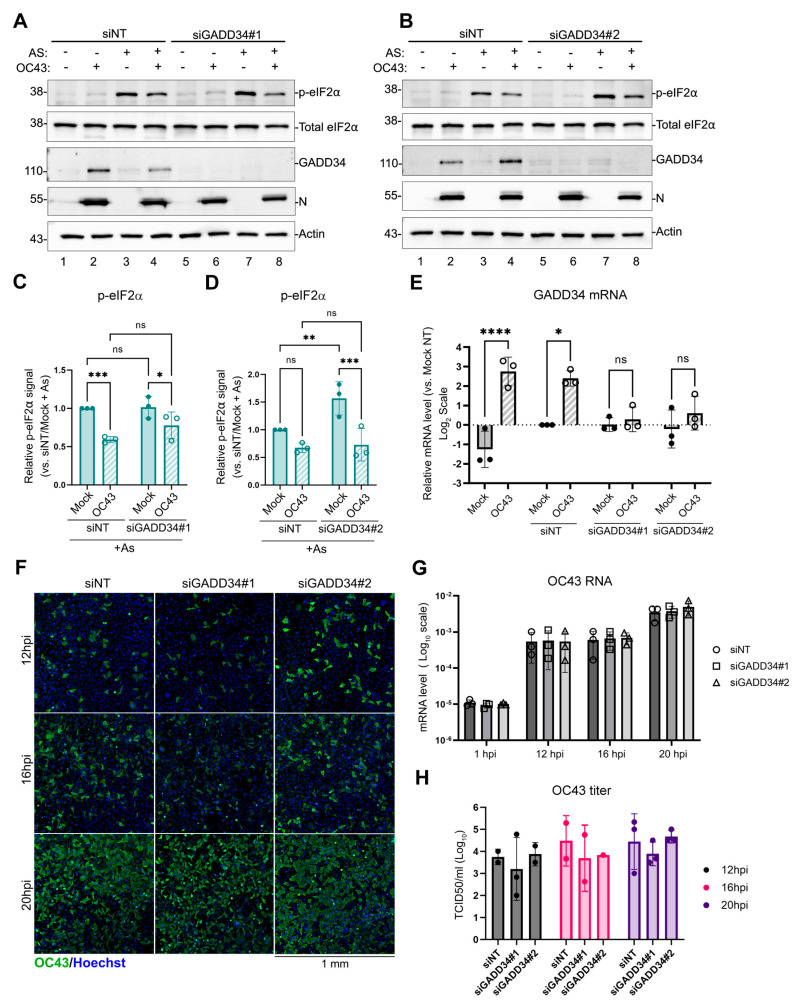
GADD34 is not responsible for the inhibition of eIF2α phosphorylation in OC43-infected cells. GADD34 expression was silenced in 293A cells using specific siRNAs (siGADD34#1 and siGADD34#2) or cells were transfected with non-targeting control siRNA (siNT). (**A**,**B**) Western blot analysis of untreated or As-treated mock- or OC43 infected cells performed at 24 hpi. (**C**,**D**) Relative level of eIF2α phosphorylation was quantified from Western blot images represented in (**A**,**B**). (**E**) Levels of GADD34 transcript were determined by RT-QPCR. (**F**) Immunofluorescence staining of OC43-infected cells pre-treated with the indicated siRNAs. Infected cells at 12, 16, and 20 hpi were visualised using the antibody to OC43 nucleoprotein (OC43, green). Nuclei were stained with Hoechst dye (blue). (**G**) Total RNA was isolated from infected cells at the indicated times post-infection and levels of OC43 genomic RNA were determined by RT-QPCR. Values were normalized to 18S. (**H**) The production of infectious virions was measured in cell supernatants. For all plots, each data point represents an independent biological replicate. Error bars = standard deviation. Two-way ANOVA and Tukey multiple comparisons tests were performed to determine statistical significance (****, *p*-value < 0.0001; ***, *p*-value < 0.001; **, *p*-value < 0.01; *, *p*-value < 0.05; ns, non-significant).

To test if GADD34 upregulation is responsible for the inhibition of As-induced eIF2α phosphorylation in OC43 infected cells, and to determine if GADD34 induction plays a role in viral replication, we silenced its expression using transfection with two different GADD34-targeting siRNAs (siGADD34#1 and siGADD34#2). As a control, we used non-targeting siRNA (siNT). After 48 h, transfected cells were mock-infected or infected with OC43 virus. At 24 hpi, GADD34 expression and As-induced eIF2α phosphorylation were analyzed using Western blotting. Both siRNAs effectively silenced OC43-induced GADD34 expression without affecting the inhibition of eIF2α phosphorylation triggered by As (Figure 5A–D). Similarly, both siRNAs decreased GADD34 mRNA levels (Figure 5E). To determine viral replication kinetics, siRNA-transfected cells were infected with OC43 at MOI ~0.1 and viral replication was assessed at 12, 16, and 20 hpi using an immunofluorescence microscopy analysis of infected cells (Figure 5F), RT-QPCR for genomic RNA levels (Figure 5G), and the release of infectious virions (Figure 5H). These analyses revealed that in our infection model, GADD34 is not required for efficient OC43 replication, despite the strong induction of this host protein by the virus.

## 4. Discussion

**Figure 6:**
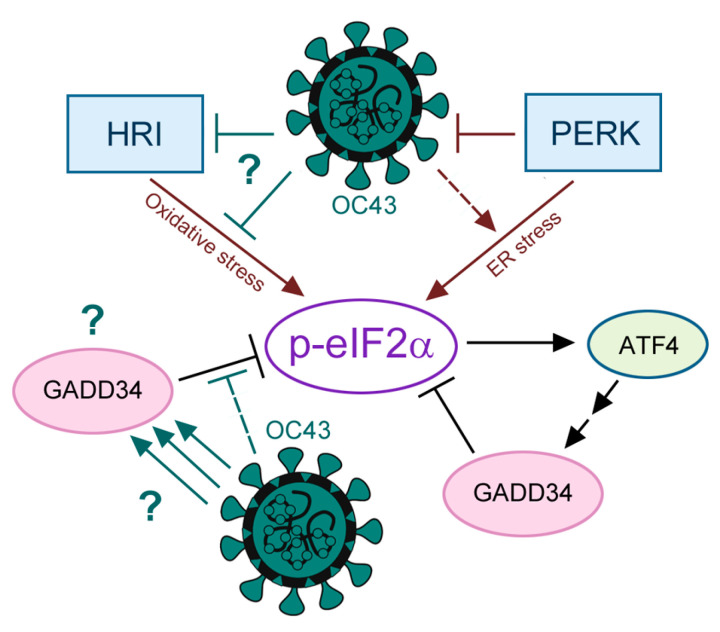
A schematic diagram representing the main findings of this work. Question marks indicate the main unanswered questions that should be addressed in future studies: How does OC43 inhibit HRI? What is the mechanism of GADD34 upregulation by OC43? What is the function of GADD34 in OC43-infected cells?

In this work, we examined some of the key aspects of the ISR pathway in 293A cells infected with human common cold virus OC43 (Figure 6). Previously, we used the same infection model to demonstrate that OC43 infection proceeds efficiently in these cells and does not trigger strong eIF2α phosphorylation or SG formation [29]. Most importantly, OC43-infected cells become less responsive to As, a strong ISR inducer that triggers robust eIF2α phosphorylation and SG formation in mock- but not in OC43-infected cells. Due to its potency in triggering eIF2α phosphorylation and translation arrest, As is often used in research labs to induce ISR-dependent SG formation [4]. We probed the response to As in infected cells for the same reason; however, the level of oxidative stress induced by 50 µM As is not physiological and only serves as a probe to reveal potential eIF2α phosphorylation inhibition. Since coronavirus mRNAs are sensitive to ISR-induced translation arrest [28], they must evolve mechanisms to suppress ISR to ensure robust viral protein synthesis and replication. To characterize the upstream activation of eIF2α kinases by OC43, we focused on PKR and PERK, the two kinases that are often activated by virus infections [9]. For example, both kinases are activated by severe acute respiratory syndrome coronavirus and chicken gammacoronavirus infectious bronchitis virus [41,42], and PERK is shown to be activated by a number of different coronaviruses and can be involved in virus-induced cell death and inflammation [28,34,43,44]. In our infection model, we did not detect PKR activation by OC43. Moreover, we did not observe the inhibition of PKR activation by poly(I:C) transfection in OC43-infected cells compared to mock. This suggests that OC43 is efficient at avoiding the detection of its dsRNA molecules by PKR by shielding them inside the double membrane replication transcription compartments and/or by the action of the Nsp15 protein [45], and does not actively block PKR activation. By contrast, OC43 infection caused a detectable activation of PERK, consistent with its ability to trigger ER stress in infected cells [33,46,47]. Interestingly, PERK activation was previously shown to either inhibit (transmissible gastroenteritis virus, [28]) or promote (porcine epidemic diarrhea virus, [44]) coronavirus replication. In our study, we demonstrate that PERK is an antiviral factor for OC43, as the silencing of PERK expression in 293A cells enhanced viral protein accumulation and infectious virion release. Interestingly, despite PERK activation, eIF2α phosphorylation levels in OC43 remained low, confirming that it is inhibited in virus-infected cells.

In our infection model, we demonstrate the robust induction of the downstream ISR effector GADD34 by OC43. GADD34 is a multifunctional protein involved in cellular stress responses. Its primary role in ISR is the recovery of protein synthesis after stress by forming a complex with PP1 to dephosphorylate eIF2α [13]. Beyond its involvement in the ISR, GADD34 plays a role in the modulation of cell death and cell cycle regulation, contributing to growth arrest under certain stress conditions [48,49,50]. The specific functions of GADD34 are context-dependent and vary based on the nature of the stress stimuli and cellular context [1,48,50]. Given robust GADD34 induction by OC43 infection, the inhibition of eIF2α phosphorylation by OC43 could be explained by GADD34-mediated recruitment of PP1 and increased eIF2α dephosphorylation rates. However, we were unable to alter the inhibition of As-induced eIF2α phosphorylation in OC43-infected cells by silencing GADD34. In mock-infected cells, Tg induced lower levels of GADD34 compared to those induced by OC43 infection, and this induction resulted in the apparent dephosphorylation of eIF2α at 4 h post-treatment compared to its peak at 1 h post-treatment. These results indicate that most of the GADD34 induced by the virus is diverted to perform other functions, but that the ISR negative feedback loop is intact in 293A cells.

The induction of GADD34 by Tg followed eIF2α phosphorylation by PERK and the production of the ATF4 transcription factor that normally functions upstream of GADD34 induction in the ISR pathway. Our analyses showed that ATF4 is not required for GADD34 induction by ER stress in 293A cells, because GADD34 protein accumulation in response to Tg or OC43 infection was unaffected by ATF4 silencing. In addition to ATF4, GADD34 can be induced by transcription factors CHOP and ATF3 [1], as well as the interferon regulatory factor 3 (IRF3) [37]. Both CHOP and ATF3 are induced by ATF4; however, other stimuli can upregulate ATF3 [51,52]. IRF3 is the main transcription factor activated by cellular sensors of viral nucleic acids and an important driver of type I and type III interferon expression [53]. OC43 was shown to activate IRF3 and induce interferon response genes in lung fibroblast cell line MRC5 [54], but in another study OC43 was demonstrated to be very efficient at avoiding type I and type III interferon induction in primary human bronchial epithelial cells [55]. Future studies will determine if ATF3, IRF3, or other transcription factors implicated in GADD34 induction [38,39] are responsible for the upregulation of GADD34 in OC43-infected cells.

To summarize, our work revealed that OC43 infection activates the PERK arm of the ISR that suppresses viral protein synthesis and replication. At the same time, OC43 robustly induces GADD34, a multifunctional protein normally involved in eIF2α dephosphorylation. However, GADD34 silencing had no effect on the viral inhibition of As-induced eIF2α phosphorylation or virus replication. For our infection model, we used 293A cells, a kidney-derived cell line that can be productively infected by OC43 [29]. The 293A cells are a clone of the human embryonic kidney 293 cell line that contains a stably integrated copy of the adenovirus E1 gene that encodes the E1a and E1b proteins. These viral proteins can potentially skew the cellular responses to OC43 infection, as they are shown to attenuate interferon induction [56,57]. Our previous work showed that eIF2α phosphorylation and SG inhibition by OC43 occurs in multiple cell types, including the untransformed immortalized human upper airway BEAS2B cells [29]. In this study, we confirmed strong GADD34 upregulation by OC43 in Vero cells that do not express adenoviral E1 gene products. However, the use of transformed cell lines deficient in type I interferon responses represents a limitation of our study, and it is possible that GADD34 may perform an important proviral or antiviral function in other infection models. Another limitation of this study is that we only tested PKR and PERK activation by OC43. Importantly, while Tg-triggered eIF2α phosphorylation by PERK was partially inhibited by OC43, PKR-mediated phosphorylation of eIF2α in response to poly(I:C) transfection was not. It is possible that the mechanism of ISR inhibition by OC43 is stress- or kinase-specific. Given the threat of ISR and eIF2α phosphorylation to coronavirus mRNA translation, future research should examine the activation of HRI and GCN2 and address whether OC43 directly inhibits eIF2α phosphorylation by HRI or decreases oxidative stress in infected cells and how these mechanisms influence virus fitness and pathogenesis.

## Data Availability

All data necessary for the interpretation of the findings presented in this work are contained within the manuscript figures.

## References

[1] Pakos-Zebrucka K., Koryga I., Mnich K., Ljujic M., Samali A., Gorman A.M. (2016). The Integrated Stress Response. EMBO Rep..

[2] Jackson R.J., Hellen C.U.T., Pestova T.V. (2010). The Mechanism of Eukaryotic Translation Initiation and Principles of Its Regulation. Nat. Rev. Mol. Cell Biol..

[3] Lu L., Han A.P., Chen J.J. (2001). Translation Initiation Control by Heme-Regulated Eukaryotic Initiation Factor 2alpha Kinase in Erythroid Cells under Cytoplasmic Stresses. Mol. Cell Biol..

[4] McEwen E., Kedersha N., Song B., Scheuner D., Gilks N., Han A., Chen J.-J., Anderson P., Kaufman R.J. (2005). Heme-Regulated Inhibitor Kinase-Mediated Phosphorylation of Eukaryotic Translation Initiation Factor 2 Inhibits Translation, Induces Stress Granule Formation, and Mediates Survival upon Arsenite Exposure. J. Biol. Chem..

[5] Wek S.A., Zhu S., Wek R.C. (1995). The Histidyl-tRNA Synthetase-Related Sequence in the eIF-2 Alpha Protein Kinase GCN2 Interacts with tRNA and Is Required for Activation in Response to Starvation for Different Amino Acids. Mol. Cell Biol..

[6] Deng J., Harding H.P., Raught B., Gingras A.-C., Berlanga J.J., Scheuner D., Kaufman R.J., Ron D., Sonenberg N. (2002). Activation of GCN2 in UV-Irradiated Cells Inhibits Translation. Curr. Biol..

[7] García M.A., Meurs E.F., Esteban M. (2007). The dsRNA Protein Kinase PKR: Virus and Cell Control. Biochimie.

[8] Harding H.P., Zhang Y., Bertolotti A., Zeng H., Ron D. (2000). Perk Is Essential for Translational Regulation and Cell Survival during the Unfolded Protein Response. Mol. Cell.

[9] McCormick C., Khaperskyy D.A. (2017). Translation Inhibition and Stress Granules in the Antiviral Immune Response. Nat. Rev. Immunol..

[10] Lee Y.-Y., Cevallos R.C., Jan E. (2009). An Upstream Open Reading Frame Regulates Translation of GADD34 during Cellular Stresses That Induce eIF2alpha Phosphorylation. J. Biol. Chem..

[11] Palam L.R., Baird T.D., Wek R.C. (2011). Phosphorylation of eIF2 Facilitates Ribosomal Bypass of an Inhibitory Upstream ORF to Enhance CHOP Translation. J. Biol. Chem..

[12] Harding H.P., Novoa I., Zhang Y., Zeng H., Wek R., Schapira M., Ron D. (2000). Regulated Translation Initiation Controls Stress-Induced Gene Expression in Mammalian Cells. Mol. Cell.

[13] Brush M.H., Weiser D.C., Shenolikar S. (2003). Growth Arrest and DNA Damage-Inducible Protein GADD34 Targets Protein Phosphatase 1 Alpha to the Endoplasmic Reticulum and Promotes Dephosphorylation of the Alpha Subunit of Eukaryotic Translation Initiation Factor 2. Mol. Cell Biol..

[14] Hartenian E., Nandakumar D., Lari A., Ly M., Tucker J.M., Glaunsinger B.A. (2020). The Molecular Virology of Coronaviruses. J. Biol. Chem..

[15] Woo P.C.Y., Huang Y., Lau S.K.P., Yuen K.-Y. (2010). Coronavirus Genomics and Bioinformatics Analysis. Viruses.

[16] Schirtzinger E.E., Kim Y., Davis A.S. (2022). Improving Human Coronavirus OC43 (HCoV-OC43) Research Comparability in Studies Using HCoV-OC43 as a Surrogate for SARS-CoV-2. J. Virol. Methods.

[17] Duguay B.A., Herod A., Pringle E.S., Monro S.M.A., Hetu M., Cameron C.G., McFarland S.A., McCormick C. (2022). Photodynamic Inactivation of Human Coronaviruses. Viruses.

[18] Kim M.I., Lee C. (2023). Human Coronavirus OC43 as a Low-Risk Model to Study COVID-19. Viruses.

[19] Shen L., Yang Y., Ye F., Liu G., Desforges M., Talbot P.J., Tan W. (2016). Safe and Sensitive Antiviral Screening Platform Based on Recombinant Human Coronavirus OC43 Expressing the Luciferase Reporter Gene. Antimicrob. Agents Chemother..

[20] Raymonda M.H., Ciesla J.H., Monaghan M., Leach J., Asantewaa G., Smorodintsev-Schiller L.A., Lutz M.M., Schafer X.L., Takimoto T., Dewhurst S. (2022). Pharmacologic Profiling Reveals Lapatinib as a Novel Antiviral against SARS-CoV-2 in Vitro. Virology.

[21] Künkel F., Herrler G. (1993). Structural and Functional Analysis of the Surface Protein of Human Coronavirus OC43. Virology.

[22] Rehwinkel J., Gack M.U. (2020). RIG-I-like Receptors: Their Regulation and Roles in RNA Sensing. Nat. Rev. Immunol..

[23] Brian D.A., Baric R.S. (2005). Coronavirus Genome Structure and Replication. Curr. Top. Microbiol. Immunol..

[24] Snijder E.J., Limpens R.W.A.L., de Wilde A.H., de Jong A.W.M., Zevenhoven-Dobbe J.C., Maier H.J., Faas F.F.G.A., Koster A.J., Bárcena M. (2020). A Unifying Structural and Functional Model of the Coronavirus Replication Organelle: Tracking down RNA Synthesis. PLoS Biology.

[25] Knoops K., Kikkert M., Worm S.H.V.D., Zevenhoven-Dobbe J.C., van der Meer Y., Koster A.J., Mommaas A.M., Snijder E.J. (2008). SARS-Coronavirus Replication Is Supported by a Reticulovesicular Network of Modified Endoplasmic Reticulum. PLoS Biology.

[26] Wolff G., Limpens R.W.A.L., Zevenhoven-Dobbe J.C., Laugks U., Zheng S., de Jong A.W.M., Koning R.I., Agard D.A., Grünewald K., Koster A.J. (2020). A Molecular Pore Spans the Double Membrane of the Coronavirus Replication Organelle. Science.

[27] Cencic R., Desforges M., Hall D.R., Kozakov D., Du Y., Min J., Dingledine R., Fu H., Vajda S., Talbot P.J. (2011). Blocking eIF4E-eIF4G Interaction as a Strategy To Impair Coronavirus Replication. J. Virol..

[28] Xue M., Fu F., Ma Y., Zhang X., Li L., Feng L., Liu P. (2018). The PERK Arm of the Unfolded Protein Response Negatively Regulates Transmissible Gastroenteritis Virus Replication by Suppressing Protein Translation and Promoting Type I Interferon Production. J. Virol..

[29] Dolliver S.M., Kleer M., Bui-Marinos M.P., Ying S., Corcoran J.A., Khaperskyy D.A. (2022). Nsp1 Proteins of Human Coronaviruses HCoV-OC43 and SARS-CoV2 Inhibit Stress Granule Formation. PLoS Pathog..

[30] Moffat J., Grueneberg D.A., Yang X., Kim S.Y., Kloepfer A.M., Hinkle G., Piqani B., Eisenhaure T.M., Luo B., Grenier J.K. (2006). A Lentiviral RNAi Library for Human and Mouse Genes Applied to an Arrayed Viral High-Content Screen. Cell.

[31] Sanjana N.E., Shalem O., Zhang F. (2014). Improved Vectors and Genome-Wide Libraries for CRISPR Screening. Nat. Methods.

[32] Ying S., Khaperskyy D.A. (2020). UV Damage Induces G3BP1-Dependent Stress Granule Formation That Is Not Driven by Translation Arrest via mTOR Inhibition. J. Cell Sci..

[33] Oda J.M., den Hartigh A.B., Jackson S.M., Tronco A.R., Fink S.L. (2023). The Unfolded Protein Response Components IRE1α and XBP1 Promote Human Coronavirus Infection. mBio.

[34] Xue M., Feng L. (2021). The Role of Unfolded Protein Response in Coronavirus Infection and Its Implications for Drug Design. Front. Microbiol..

[35] Sidrauski C., Acosta-Alvear D., Khoutorsky A., Vedantham P., Hearn B.R., Li H., Gamache K., Gallagher C.M., Ang K.K.-H., Wilson C. (2013). Pharmacological Brake-Release of mRNA Translation Enhances Cognitive Memory. eLife.

[36] Sidrauski C., Tsai J.C., Kampmann M., Hearn B.R., Vedantham P., Jaishankar P., Sokabe M., Mendez A.S., Newton B.W., Tang E.L. (2015). Pharmacological Dimerization and Activation of the Exchange Factor eIF2B Antagonizes the Integrated Stress Response. eLife.

[37] Clavarino G., Cláudio N., Couderc T., Dalet A., Judith D., Camosseto V., Schmidt E.K., Wenger T., Lecuit M., Gatti E. (2012). Induction of GADD34 Is Necessary for dsRNA-Dependent Interferon-β Production and Participates in the Control of Chikungunya Virus Infection. PLoS Pathog..

[38] Haneda M., Xiao H., Hasegawa T., Kimura Y., Nakashima I., Isobe K. (2004). Regulation of mouse GADD34 gene transcription after DNA damaging agent methylmethane sulfonate. Gene.

[39] Gambardella G., Staiano L., Moretti M.N., De Cegli R., Fagnocchi L., Di Tullio G., Polletti S., Braccia C., Armirotti A., Zippo A. (2020). GADD34 is a modulator of autophagy during starvation. Sci. Adv..

[40] Walter P., Ron D. (2011). The Unfolded Protein Response: From Stress Pathway to Homeostatic Regulation. Science.

[41] Liao Y., Fung T.S., Huang M., Fang S.G., Zhong Y., Liu D.X. (2013). Upregulation of CHOP/GADD153 during Coronavirus Infectious Bronchitis Virus Infection Modulates Apoptosis by Restricting Activation of the Extracellular Signal-Regulated Kinase Pathway. J. Virol..

[42] Krähling V., Stein D.A., Spiegel M., Weber F., Mühlberger E. (2009). Severe Acute Respiratory Syndrome Coronavirus Triggers Apoptosis via Protein Kinase R but Is Resistant to Its Antiviral Activity. J. Virol..

[43] Zhou Y., Zhang Y., Dong W., Gan S., Du J., Zhou X., Fang W., Wang X., Song H. (2023). Porcine Epidemic Diarrhea Virus Activates PERK-ROS Axis to Benefit Its Replication in Vero E6 Cells. Vet. Res..

[44] Keramidas P., Papachristou E., Papi R.M., Mantsou A., Choli-Papadopoulou T. (2023). Inhibition of PERK Kinase, an Orchestrator of the Unfolded Protein Response (UPR), Significantly Reduces Apoptosis and Inflammation of Lung Epithelial Cells Triggered by SARS-CoV-2 ORF3a Protein. Biomedicines.

[45] Gao B., Gong X., Fang S., Weng W., Wang H., Chu H., Sun Y., Meng C., Tan L., Song C. (2021). Inhibition of Anti-Viral Stress Granule Formation by Coronavirus Endoribonuclease Nsp15 Ensures Efficient Virus Replication. PLoS Pathog..

[46] Nguyen L.C., Renner D.M., Silva D., Yang D., Parenti N.A., Medina K.M., Nicolaescu V., Gula H., Drayman N., Valdespino A. (2022). SARS-CoV-2 Diverges from Other Betacoronaviruses in Only Partially Activating the IRE1α/XBP1 Endoplasmic Reticulum Stress Pathway in Human Lung-Derived Cells. mBio.

[47] Favreau D.J., Desforges M., St-Jean J.R., Talbot P.J. (2009). A Human Coronavirus OC43 Variant Harboring Persistence-Associated Mutations in the S Glycoprotein Differentially Induces the Unfolded Protein Response in Human Neurons as Compared to Wild-Type Virus. Virology.

[48] Song P., Yang S., Hua H., Zhang H., Kong Q., Wang J., Luo T., Jiang Y. (2019). The Regulatory Protein GADD34 Inhibits TRAIL-Induced Apoptosis via TRAF6/ERK-Dependent Stabilization of Myeloid Cell Leukemia 1 in Liver Cancer Cells. J. Biol. Chem..

[49] Farook J.M., Shields J., Tawfik A., Markand S., Sen T., Smith S.B., Brann D., Dhandapani K.M., Sen N. (2013). GADD34 Induces Cell Death through Inactivation of Akt Following Traumatic Brain Injury. Cell Death Dis..

[50] Shi W., Sun C., He B., Xiong W., Shi X., Yao D., Cao X. (2004). GADD34-PP1c Recruited by Smad7 Dephosphorylates TGFbeta Type I Receptor. J. Cell Biol..

[51] Ku H.-C., Cheng C.-F. (2020). Master Regulator Activating Transcription Factor 3 (ATF3) in Metabolic Homeostasis and Cancer. Front. Endocrinol..

[52] Rohini M., Haritha Menon A., Selvamurugan N. (2018). Role of Activating Transcription Factor 3 and Its Interacting Proteins under Physiological and Pathological Conditions. Int. J. Biol. Macromol..

[53] Dalskov L., Gad H.H., Hartmann R. (2023). Viral Recognition and the Antiviral Interferon Response. EMBO J..

[54] Duncan J.K.S., Xu D., Licursi M., Joyce M.A., Saffran H.A., Liu K., Gohda J., Tyrrell D.L., Kawaguchi Y., Hirasawa K. (2023). Interferon Regulatory Factor 3 Mediates Effective Antiviral Responses to Human Coronavirus 229E and OC43 Infection. Front. Immunol..

[55] Loo S.-L., Wark P.A.B., Esneau C., Nichol K.S., Hsu A.C.-Y., Bartlett N.W. (2020). Human Coronaviruses 229E and OC43 Replicate and Induce Distinct Antiviral Responses in Differentiated Primary Human Bronchial Epithelial Cells. Am. J. Physiol. Lung Cell Mol. Physiol..

[56] Kalvakolanu D.V., Bandyopadhyay S.K., Harter M.L., Sen G.C. (1991). Inhibition of interferon-inducible gene expression by adenovirus E1A proteins: Block in transcriptional complex formation. Proc. Natl. Acad. Sci. USA.

[57] Chahal J.S., Qi J., Flint S.J. (2012). The human adenovirus type 5 E1B 55 kDa protein obstructs inhibition of viral replication by type I interferon in normal human cells. PLoS Pathog..

